# Extraction of Iron from the Rabbit Anterior Chamber with Reverse Iontophoresis

**DOI:** 10.1155/2015/425438

**Published:** 2015-07-15

**Authors:** Shoubin Sun, Huijie Diao, Fali Zhao, Jianhai Bai, Yuan Zhou, Hao Cui, Liqiong Zhang

**Affiliations:** ^1^Department of Ophthalmology, The First Affiliated Hospital of Harbin Medical University, Harbin, Heilongjiang 150001, China; ^2^Department of Ophthalmology, Affiliated Hongqi Hospital, Mudanjiang Medical College, Mudanjiang, Heilongjiang 157000, China; ^3^Department of Cardiology, The First Affiliated Hospital of Harbin Medical University, Harbin, Heilongjiang 150001, China

## Abstract

Ocular siderosis is a common eye disease caused by retention of an iron-containing intraocular foreign body in the eye. Iron-containing intraocular foreign bodies may cause severe inflammatory reaction and affect visual function. Currently the optimal treatment method of ocular siderosis is a moot point. This study used the reverse iontophoresis technique to noninvasively extract iron from the rabbit anterior chamber. By slit lamp observation and histological examination, reverse iontophoresis treatment has a good effect on ocular siderosis. Reverse iontophoresis seems to be a noninvasive and promising approach to extract iron from the anterior chamber to treat ocular siderosis.

## 1. Introduction

Intraocular foreign body is a special kind of eye injury that has greater harm compared to the general penetrating eye injuries. In addition to the injury caused by mechanical damage, foreign bodies in the eye can also lead to increased eye damage due to its retention. In particular, iron-containing intraocular foreign bodies (IOFB) may cause severe inflammatory reaction and affect visual function [1]. This disease is called ocular siderosis [2]. Clinical manifestations of anterior segment in ocular siderosis include bullous keratopathy, corneal neovascularization, iris heterochromia, mydriasis, cataract, lens subluxation, uveitis, and secondary glaucoma [3, 4]. Posterior segment manifestations in ocular siderosis include retinal detachment, pigmentary degeneration of the retina, cotton wool spots in fluorescein angiography (FA), fluorescein leakage, and local capillary nonperfusion. Electroretinography (ERG) is higher than the normal reaction at first but will reduce with disease progression [5–7]. Ocular siderosis can cause irreversible retinal injuries and visual function damage and can even cause eyeball loss in the patients [8]. Previous studies have shown that the damage of ocular siderosis is mainly caused by oxidative stress from iron toxicity [9, 10]. Iron oxidation is majorly based on Fenton reaction: iron can react with hydrogen peroxide, superoxide, or other reactive oxygen intermediates to produce reactive oxygen species such as hydroxyl radicals. These molecules can cause a cascade of damage and destroy the cell membrane phospholipids, proteins, and nucleic acids, which will lead to cell lesions and even death. In order to save a patient's visual function, it is important to remove the foreign body as soon as possible. Currently the optimal treatment method of ocular siderosis is a moot point. The best time for the surgery and the prognosis for maintenance or extraction of foreign bodies are both uncertain. Also, there will be a certain degree of surgical trauma [11]. Iron chelators such as deferoxamine (DFO, Desferal) therapy are valuable. However, the limited effect and high cost restrict its usage [12, 13]. Therefore, the development of a noninvasive method to extract iron from an intraocular foreign body is a pressing clinical need.

Iontophoresis is a noninvasive technique in which a low electric current is applied to enhance ionized drug penetration into tissue [14]. The central tenet of iontophoresis is simply the basic electrical principle where oppositely charged ions attract and similarly charged ions repel. Ionized drug substance through the anode (for a positively charged drug) or the cathode (for a negatively charged drug) under the action of the current will penetrate into tissue [15]. Iontophoresis technology is used to direct therapeutic drugs to target sites with the advantages of being easy to use and safe, with limited systemic side effects [16]. At present, the technology has been successfully applied to various clinical diseases, especially in the treatment of hyperhidrosis [17]. In the past few decades, iontophoresis has been extensively studied for the delivery of drugs to treat eye diseases [18–21].

Iontophoresis can be used in two directions—not only driving ions into the skin, but also removing ions from the body. Thus, this method can be used to obtain chemical information from the body, which makes the clinical tests carried out without blood possible. Reverse iontophoresis (RI) is a technique that can be used to noninvasively extract polar substances through the skin by using a low electric current [22]. The mechanism of extraction involves either electromigration of charged species to the electrode of opposite polarity or electroosmosis of neutral molecules to the cathode or anode, or a combination of these two processes [23]. RI has been successfully employed in the GlucoWatch Biographer to continuously monitor blood glucose [24, 25]. Also RI has been investigated for monitoring and extracting endogenous metabolites, such as urea [23, 26], amino acids [27, 28], prostaglandin E_2_ [29], uric acid [30], L-lactic acid [31], or drugs (gabapentin [32], amikacin [33], and lithium [34]). It has been demonstrated that RI can be used to extract iron from cornea in* in vitro* studies [35]. However, the use of RI to extract the intraocular drug or ions* in vivo* has not been reported so far.

Because iron foreign body within the eye or ocular siderosis of iron is positively charged, we hypothesized that they can be extracted out of the body by electromigration or electroosmosis. Therefore, the purpose of this study is to extract iron from the anterior chamber of the cornea. This may provide a noninvasive, economical, and practical method for the treatment of iron foreign body inside the eye with few systemic side effects.

## 2. Materials and Methods

### 2.1. Instruments and Reagents

The main instruments used in this study include slit lamp microscope (Topcon SL-1E, Topcon, Japan); inductively coupled plasma atomic emission spectroscopy (ICP-OES, Optima 5300DV, Perkin Elmer, USA); drug iontophoresis apparatus (Xi'an Huaya Product Company, China); eyecup (MEDA Co., Ltd., Tianjin, China) which is made of polycarbonate, inner diameter of 12 mm; Ag/AgCl electrodes (Aida Hengsheng, Tianjin, China); and 250 *μ*L Hamilton syringe fitted with 33 G needle (Hamilton, Bonaduz, Switzerland). Soluble Ferric pyrophosphate (FPP) was purchased from Sigma-Aldrich Corporation (USA) and 0.4% Oxybuprocaine Hydrochloride Eye Drops was from Santen (Osaka, Japan). Other chemicals and reagents were purchased from Sigma and were of the highest grades available. Sterile deionized water (resistivity ≥18 MΩ cm) was used to prepare all solutions. All surgical instruments were nickel-chromium alloy equipment. For HPLC solvent preparation, distilled water and HPLC-grade solvents were used.

### 2.2. Animals

Forty New Zealand White Rabbits (male and female, 2.5~3.0 kg) were provided by the Animal Center of Harbin Medical University. These animals were given a standard diet, with free access to water, and were placed in 25 ± 1°C, 50 ± 5% relative humidity and 12 hours of light per day. All experiments were carried out under veterinary supervision following approval of the protocols by the Ethics Committee of Harbin Medical University. No adverse reactions were observed after one week of adaptive feeding. No anterior segment and fundus disease was observed by slit lamp microscope and direct ophthalmoscopy examination. Group I is the control group which includes eight rabbits (16 eyes) without modeling. The right eyes (8) were used to measure the iron concentration in the liquid receiving tank, whereas the left eyes were given RI for 15 min at 0.4 mA current to determine RI corneal safety. 32 rabbits were underwent right anterior manufacture iron foreign body model (monocular modeling), and then randomly divided into II, III, IV and V four groups of eight eyes to perform RI experiment.

### 2.3. Anterior Chamber Iron Foreign Body Model

This experiment was performed as described previously [36]. Rabbits were anesthetized with 1% pentobarbital sodium through the ear vein and disinfected with 5% povidone iodine. Surgery eyes were open with speculum and conjunctival sacs were rinsed with 0.9% normal saline for three minutes. One drop of 0.4% oxybuprocaine chlorydrate was instilled three times every 10 minutes. A 33 G needle fitted on 250 *μ*L Hamilton syringe was used to penetrate into the anterior chamber of rabbit eyes and stopped within 1 mm from the temporal limbus to avoid touching the corneal endothelium, iris, and lens. A volume of 0.1 mL liquid was extracted and then 0.1 mL FPP solution (25 mg/mL) was immediately injected into the anterior chamber to produce the iron foreign body model. Slit lamp microscope was used to check the success of the model.

### 2.4. RI Experiment

**Figure 1:**
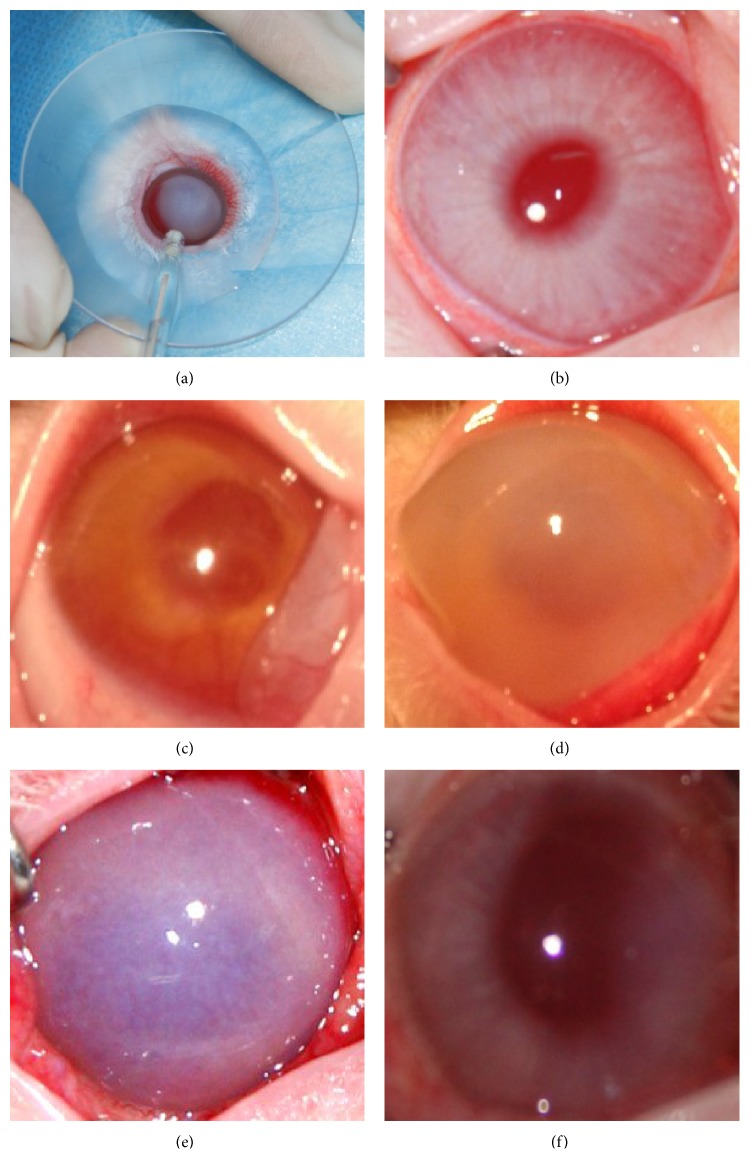
Anterior segment photographs. (a) RI experiment cathode electrode and the eyecup. The cup is placed around eye cornea, and Ag/AgCl electrode is inserted into the eyecup to provide the cathode current for RI experiments. (b) The normal rabbit eye with smooth and transparent cornea, and transparent anterior chamber in the control group. (c) Immediate image after anterior chamber iron foreign body model. The anterior chamber was brown, and oozing around the pupil was observed. (d) 24 h after anterior iron foreign body model, there were conjunctival hyperemia, corneal edema, large bullous keratopathy, and rust-colored pigmentation, and the anterior chamber was brown. (e) Six days after modeling with 0 mA current RI. (f) Six days after modeling with 0.4 mA current RI.

The RI device is a DC power supply with two electrodes and is processed by the drug iontophoresis apparatus. It has an accuracy of ±0.01 msec on timing and ±0.01 *μ*A on delivering current. An eyecup with 12 mm inner diameter (diffusion surface area of 1.13 cm^2^) was placed around the rabbit cornea and was mounted with 3 mL 0.9% NaCl solution to use as accepting pool. An Ag/AgCl electrode was inserted into the eye as a cathode (Figure 1(a)). The anode was attached to the ear of the animal, as close as possible to the former electrode, to obtain minimal resistance. Ferrous ions were extracted from the anterior chamber through the cornea into the accepting pool. Groups II, III, IV, and V were given different constant current strength, 0 mA, 0.4 mA (0.35 mA/cm^2^), 0.3 mA (0.27 mA/cm^2^), and 0.2 mA (0.18 mA/cm^2^), to the cornea using the RI device for 15 min. Liquid collected by the eye cup was injected into a 5 mL Eppendorf tube and stored in the freezer at −20°C until analysis. After 24 hours of the RI modeling experiment, iron concentrations in the export liquid of groups I, IV, and V were determined. After 24 h, 2 d, 3 d, 4 d, 5 d, and 6 d of the RI experiment, iron ion concentrations were determined and gross changes were examined with the eye slit lamp in groups II and III to detect the daily changes.

### 2.5. Aqueous Humor Sample Collection

After RI experiment, rabbits aqueous humor in groups I, II, and III was collected. Through standard corneal paracentesis [37], aqueous humor was aspirated using a 30-gauge cannula on a BD Insulin syringe (1 mL). Meticulous care was taken to avoid touching the iris, lens, corneal endothelium, and conjunctiva. A volume of 0.2 mL aqueous humor was collected and was immediately transferred to an Eppendorf tube and frozen in liquid nitrogen. Samples were then stored at −80°C and kept protected from light until the iron analysis was performed.

### 2.6. Histological Examination

After collection of aqueous humor sample, rabbits from groups I, II, and III were immediately euthanized with excess 1% pentobarbital sodium. Complete corneal tissues were collected and fixed in 10% formalin for 24 hours. Paraffin blocks were prepared and 5 *μ*m thick sections were subjected to hematoxylin and eosin (H&E) staining [38]. Eye sections were also performed with Prussian blue staining to detect ferric iron [8].

### 2.7. Iron Concentration Determination

Quantitative iron measurement was performed to detect the concentration of iron in the receiving pool using ICP-OES [39]. The ICP-OES spectrometer was used with the following parameters: frequency, 40.68 MHz, demountable quartz torch; FR power, 1300 kW; plasma gas (Ar) flow, 15 L/min; auxiliary gas (Ar) flow, 0.2 L/min; nebulizer gas (Ar) flow, 0.8 L/min; observation height, 15 mm; nebulizer, quick removal of corrosion spider-type nebulizer; sample pump flow rate, 1.5 mL/min; viewing configuration, axial; and wavelength Fe, 238.204 nm.

### 2.8. Statistical Analysis

Statistical analysis was performed with SPSS17.0. Quantitative data were presented as mean ± standard deviation for statistical description; groups were compared using *t*-test or analysis of variance, multiple comparisons using the SNK test, regression analysis using the general linear regression analysis, data from multiple time points data were analyzed using repeated variance; all tests were two-sided test. *p* < 0.05 was considered as statistically significant.

## 3. Results

### 3.1. Slit Lamp Examination

No abnormality of the cornea was observed for control group I (Figure 1(b)). Immediately after iron foreign body modeling the slit lamp microscope examination indicated that anterior chamber was brownish yellow, no bubbles and aqueous leakage, and a small amount of membrane surrounding the pupil was observed (Figure 1(c)). Conjunctival hyperemia, corneal epithelium and endothelial edema, bullous keratopathy, brownish yellow anterior chamber, slightly dilated pupil, pigmentation in the anterior lens, no coloring by corneal fluorescein staining, and no aqueous leakage were observed at 24 hours after modeling and before RI (Figure 1(d)). Six days after modeling with 0 mA current RI, conjunctival hyperemia, corneal edema, diffuse inflammatory infiltration shaped mist muddy, muddy anterior chamber aqueous humor, and mydriasis were observed (Figure 1(e)). Six days after modeling with 0.4 mA current RI, mild conjunctival hyperemia, transparent cornea, mildly dilated pupil, and no pigmentation in anterior chamber lens were observed (Figure 1(f)).

### 3.2. Iron Concentration in Liquid Receiving Tank Is Increasing with Current Intensity of RI

**Figure 2:**
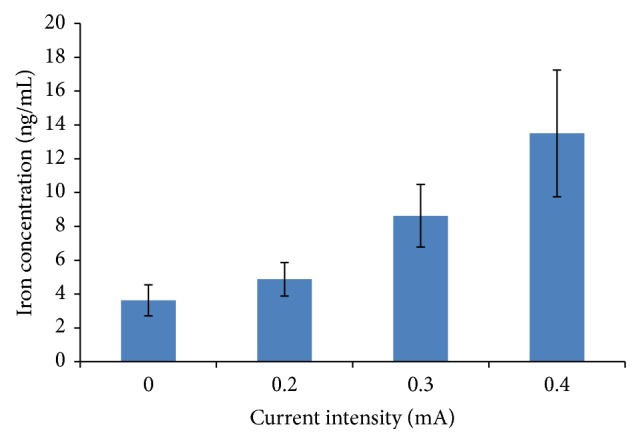
Iron concentrations of collected liquid following one-time RI with different current intensities.

The iron concentration in liquid from the control group (group I) and the modeling group without RI (group II) was 2.88 ± 0.83 ng/mL and 3.63 ± 0.92 ng/mL, respectively. The iron concentration in group II was higher than that in control group I, but the difference was not statistically significant. With the increase of the current intensity from 0 mA, 0.2 mA, and 0.3 mA to 0.4 mA in RI experiment, the collected liquid iron concentrations were significantly increased from 3.63 ± 0.92 ng/mL, 4.88 ± 0.99 ng/mL, and 8.63 ± 1.85 ng/mL to 13.5 ± 3.74 ng/mL (Figure 2).

### 3.3. Iron Concentration in Liquid Receiving Tank Is Decreasing by Repeated RI

**Figure 3:**
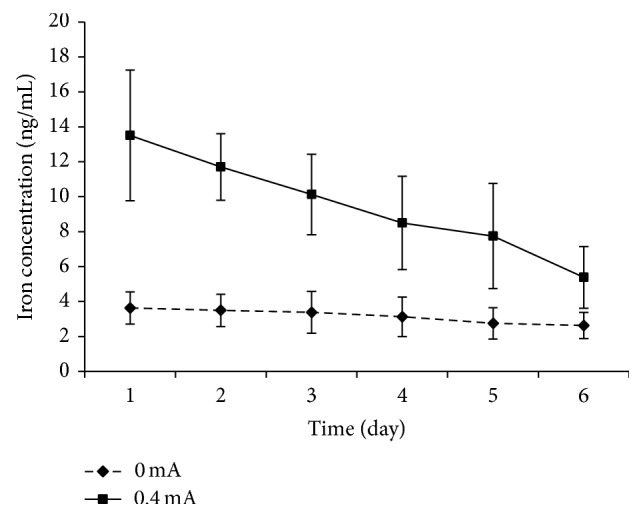
Iron concentrations of collected liquid at different time points following RI.

Groups II (0 mA) and III (0.4 mA) were performed with repeated RI on 24 h, 2 d, 3 d, 4 d, 5 d, and 6 d after modeling with the change in time, and the iron concentration in the collected liquid was not significantly changed in group I, but it was gradually decreasing in group III (Figure 3).

### 3.4. Iron Concentration in the Aqueous Humor Is Decreased by RI in the Modeling Group

The iron concentration in the aqueous humor from control group was 0.35 ± 0.31 *μ*g/mL. It was greatly increased to 10.72 ± 3.23 *μ*g/mL in the modeling group with 0 mA current RI. However, the iron concentration was dramatically decreased to 3.39 ± 2.89 *μ*g/mL in the modeling group with 0.4 mA current RI.

### 3.5. Histological Examination

**Figure 4:**
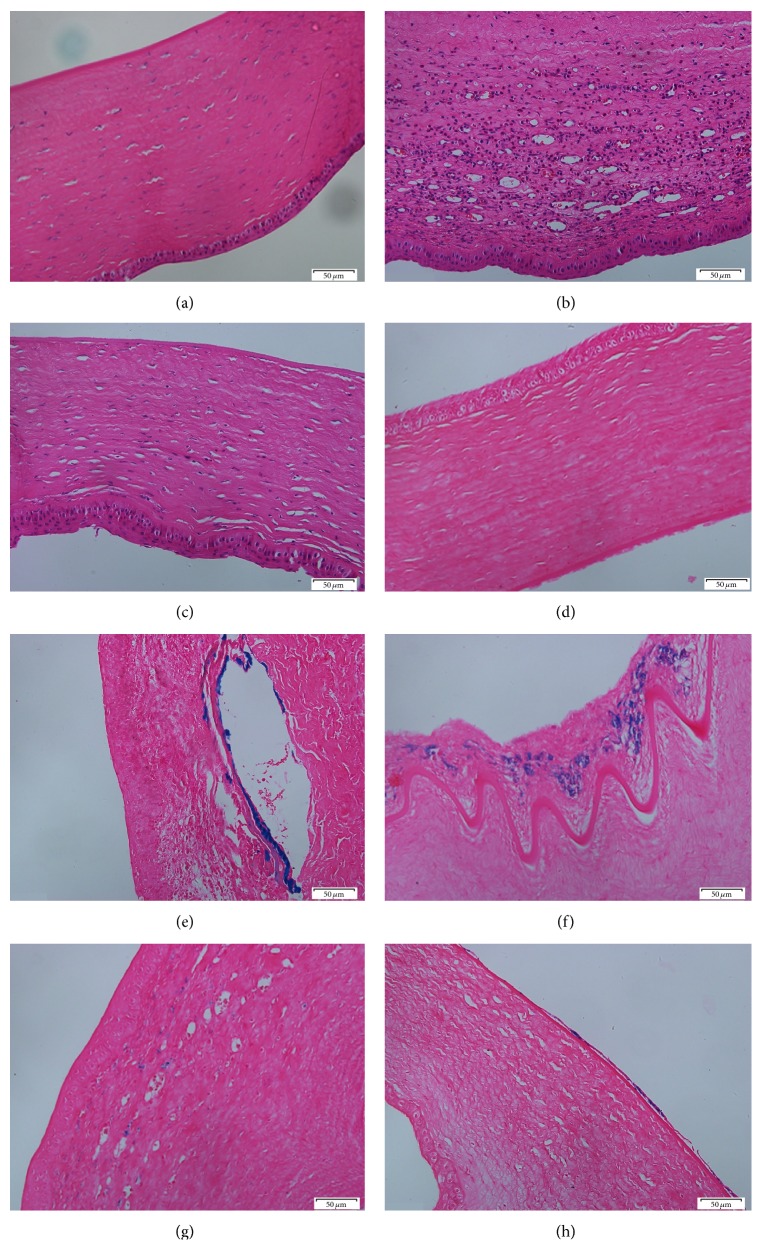
Histological examination of cornea. Hematoxylin and eosin (H&E) staining of cornea from control group (a) and the iron-containing IOFB modeling group with 0 mA (b) and 0.4 mA RI treatment (c). Prussian blue iron staining of cornea from normal control group (d) and the iron-containing IOFB modeling group with 0 mA (e and f) and 0.4 mA RI treatment (g and h). (Original magnification, 20x. Scale bar, 50 *μ*m.)

Hematoxylin and eosin staining indicated that stromal cells were neatly arranged, and no inflammatory cell infiltration was observed in the normal control group I (Figure 4(a)). Swelling corneal epithelium, disordered corneal stroma, a large number of neutrophils, lymphocytes and eosinophils infiltration, and more fibroblasts and neovascularization within the matrix were observed in the modeling group (Figure 4(b)). However, tightly packed layers of the cornea with only a small amount of inflammatory cell infiltration and mild disorder in matrix fibers were observed in the RI treatment group (Figure 4(c)).

No iron particle deposition in cornea was observed by Prussian blue staining in the normal control group (Figure 4(d)). Blue clusters of aggregated iron particles are deposited on corneal stroma and a large number of blue iron particles corneal adhesion to endothelial cells were observed in the modeling group (Figures 4(e) and 4(f)). In the RI treatment group only a small amount of blue iron particles scattered on the corneal stroma and corneal endothelium can be observed (Figures 4(g) and 4(h)).

## 4. Discussion

Ocular siderosis is a common eye disease caused by retention of an iron-containing intraocular foreign body (IOFB) in the eye. Severe intraocular inflammation will occur in the early phase after the iron foreign body entering into the eye. Under humid environment, the iron foreign body rusts relatively quickly within the eye, leading to serious damage to the eye tissue. In the previous study, FeCl_2_, FeCl_3_ and other forms of iron were injected into the eye to form the IOFB model [40]. In the present study, we used the soluble ferric pyrophosphate (FPP) to produce iron in the IOFB model. FPP as an iron source has been proven to be safe for systemic administration [41]. After injecting 2.5 mg soluble FPP solution into the anterior chamber, the iron concentration in the aqueous humor rapidly increased, resulting in corneal edema, brownish yellow aqueous, bullous keratopathy, mydriasis, and other symptoms observed in ocular siderosis (Figures 1(c) and 1(d)). The modeling method is simple and can quickly increase the iron concentration in anterior chamber of the eye, thus shortening the study period compared to other experimental iron implantation models.

RI involves transdermal application of a low current (<0.5 mA/cm^2^) to enhance the passage of ions and uncharged polar molecules across the skin [42]. The major mechanisms for transport of ions and molecules are electromigration of charged species to the electrode of opposite polarity and electroosmosis of neutral molecules to the cathode or anode, or a combination of these two processes [26]. A previous study has confirmed that the positively charged particles hemosiderin (iron or ferrous ions) may be extracted from the cornea by RI* in vitro* [35]. In the present study, we inserted a cathode electrode into an eyecup and give different low currents for 15 min. Under the action of RI, the positively charged iron ions in the anterior chamber flow towards the cathode and enter the eyecup. This study demonstrated the safety and tolerability of RI using a current of 0.4 mA in rabbit corneas for 15 min. To be noted, some other studies even used a current intensity as high as 1.5 mA for 20 min and no adverse effects were reported [43]. These data suggest that the use of effective low current intensities to extract iron is safe and feasible.

In our study, the collected liquid iron concentrations were 2.88 ± 0.83 ng/mL and 3.63 ± 0.92 ng/mL in the control group and the modeling group with 0 mA current in RI, respectively. The basal iron may be derived from the tear. After RI experiment the iron concentration in the aqueous humor of the control group was 0.35 ± 0.31 *μ*g/mL, which is similar to the previous report [44]. The iron concentrations of aqueous humor from modeling group and the RI treatment group were 10.72 ± 3.23 *μ*g/mL and 3.39 ± 2.89 *μ*g/mL. These results suggest that RI can extract iron from the anterior chamber to the outside through the cornea, decreasing the iron concentration in the anterior chamber. The RI extracted iron concentration was proportional to the given current intensity, consistent with previously reported work showing that an increase in current intensity proportionately enhanced the extraction of gabapentin through the skin [32]. Over time, due to metabolism and repeated RI, the iron concentration in the anterior chamber was reduced so that the extracted iron by RI in the collected liquid constantly decreased.

By slit lamp observation, anterior segment of rabbit eyes from RI treated group was significantly better than the untreated control group. Hematoxylin and eosin staining also indicated that RI treated group has less inflammatory cell infiltration and more preserved matrix fibers than the modeling group. Accordingly, Prussian blue staining indicated that RI treated rabbit eyes have less iron particle deposition in the stroma and endothelium than the untreated group. These results suggest that RI treatment has a good effect on ocular siderosis. This is consistent with the previous report that the iron in the eye has a direct relationship with the prognosis of siderosis [45].

In summary, RI is a noninvasive and safe technique that can be reliably used to extract iron out of the anterior chamber of rabbit eyes. Further studies in human are required to advance this technique into clinical practice.
